# A Lamping U-Shaped Fiber Biosensor Detector for MicroRNA

**DOI:** 10.3390/s20051509

**Published:** 2020-03-09

**Authors:** Hsin-Yi Wen, Chun-Wei Huang, Yu-Le Li, Jing-Luen Chen, Yao-Tsung Yeh, Chia-Chin Chiang

**Affiliations:** 1 Department of Mechanical Engineering, National Kaohsiung University of Science and Technology, 415 Chien Kung Road, Kaohsiung 80778, Taiwan; hywen@nkust.edu.tw (H.-Y.W.); cwhuang@kuas.edu.tw (C.-W.H.); love02111110@gmail.com (Y.-L.L.); ChineseTaipeibasketball@gmail.com (J.-L.C.); 2 Department of Medical Laboratory Science and Biotechnology, Fooyin University, Kaohsiung 83102, Taiwan; glycosamine@yahoo.com.tw

**Keywords:** microRNAs detection, U-shaped optical fiber, silanization, biosensor

## Abstract

This study presents a U-shaped optical fiber developed for a facile application of microRNA detection. It is fabricated by the lamping process and packaged in a quartz tube to eliminate human negligence. In addition, silanization and electrostatic self-assembly are employed to bind gold nanoparticles and miRNA-133a probe onto the silicon dioxide of the fiber surface. For Mahlavu of hepatocellular carcinoma (HCC), detection is determined by the wavelength shift and transmission loss of a U-shaped optical fiber biosensor. The spectral sensitivity of wavelength and their coefficient of determination are found at −218.319 nm/ ng/mL and 0.839, respectively. Concurrently, the sensitivity of transmission loss and their coefficient of determination are found at 162.394 dB/ ng/mL and 0.984, respectively. A method for estimating the limit of detection of Mahlavu is at 0.0133 ng/mL. The results show that the proposed U-shaped biosensor is highly specific to miRNA-133a and possesses good sensitivity to variations in specimen concentration. As such, it could be of substantial value in microRNA detection.

## 1. Introduction

MicroRNAs (miRNAs) are small non-coding RNAs which are approximately 22 nucleotides in length [1] and play important regulatory roles in animals and plants by targeting mRNAs for cleavage or translational repression [2]. In humans, over 1000 miRNAs have been identified, and each miRNA potentially represses hundreds of target mRNAs [3]. Over the last several decades, there have been numerous studies that have found associations between miRNAs and various types of cancer, such as lymphocytic leukemia [4,5], lung cancer [6,7], colorectal neoplasia [8], Burkitt lymphoma [9], glioblastoma [10], tumor cell [11], B cell lymphomas of hepatocellular carcinoma (HCC) [12], oral squamous cell carcinoma [13], and breast cancer [14]. Relatedly, many biological techniques have been developed for miRNA detectors, including Northern blot [15], real-time PCR [16], oligonucleotide microarray [17,18], laser-induced fluorescence [19], microarray [20,21], and even various label-free techniques [22], as well as some strategy approaches for the detection of miRNAs, such as electrochemical, photoelectrochemical and optical fiber sensing methods. The purpose of this study is to review the literature on photoelectrochemical biosensors for miRNA assay that show potential to be applied in bioanalysis research. In 2019, Chang, J., et al. [23] proposed a homogeneous electrochemical biosensor for simultaneous detection of multiple tumor biomarkers based on the functionalized metal–organic frameworks (MOFs). Hou, T., et al. [24] demonstrated an immobilization-free diffusivity-mediated PEC bionsensing strategy for microRNA assay, using methylene blue in solution as the photoactive probe, and nonmodified indium tin oxide glass as the working electrode. Wang, Y., et al. [25] proposed a photoelectrochemical biosensor that was constructed on the basis of a sensitization strategy of doxorubicin sensitized graphitic carbon nitride for the ultrasensitive detection of microRNA-141. This strategy exhibits excellent specificity with the advantages of simplicity, rapidness, and good reproducibility.

Optical fiber possesses the advantages of being light in weight, small in size, and high in stability and sensitivity. It is free from electromagnetic interference and can perform single-mode fiber (SMF) multipoint measurements. With the advancement of optical fiber technology, the application of optical fiber sensors, especially in biomedicine, is becoming more and more popular. Chen et al. [26] demonstrated a dual-peak long-period fiber grating (LPFG) optical biosensor for the detection of DNA hybridization. The glass surface of the fiber was immersed into 10% 3-aminopropyl-triethoxysilane (APTES) for silanization that subsequently served as a bridge to bind probe DNA. Experimental results for the sensor showed that the drift of the wavelength and the change of transmission loss were 0.254 nm and 1.3 dB, respectively, after the LPFG sensor was incubated in 1 μM of probe DNA and that a wavelength shift occurred during the hybridization process, with an increase of 715 pm. Their results showed a maximum sensitivity of 0.254 nm/µM. Sozzi et al. [27] presented an LPFG used as a DNA biosensor to perform real-time and label-free detection. The fiber was functionalized with peptide nucleic acid (PNA) that would match with the DNA target strands. A wavelength shift of 1.2 nm was measured for a 120 nm DNA solution. Candiani et al. [28] developed an out cladding sensitized label-free DNA biosensor based on tilted fiber Bragg grating (TFBG). A double tilted fiber Bragg grating forming a modified Fabry–Perot core-cladding closed-loop cavity was functionalized with PNA probes to bind the target DNA. Their results showed a detection limit of 10 nM and detection range of 10 to 100 nM. The real-time spectral measurement results showed that a 10 nM DNA solution induced a 10% modulation of the visibility of the corresponding fringe. Huang et al. [29] presented an optical microfiber taper interferometer for the in situ detection of unlabeled single-stranded DNA targets. The taper interferometer was coated by a highly ordered pore array conjugated polymer which served as tentacles to catch single-stranded DNA molecules through π–π conjugated interaction. The refractive index (RI) information was then translated into the wavelength shift of the interference fringe. The sensor exhibited a DNA concentration sensitivity of 2.393 nm/log M and the lowest detection ability of 10^−10^ M or even lower. Their results showed a detection limit of 0.1 nM and detection range of 1 nM to 1 µM. Hsu et al. [30] demonstrated a polarization maintaining (PM) fiber constructed with spectral interferometry-based surface plasmon resonance (SPR) to detect miRNA-21 sequences. This thiol-labeled miRNA-21 DNA used as a probe was prepared in autoclaved deionized (DI) water and immobilized onto a precleaned Au surface for 8500 s at 25 °C. Then, an interferogram near the wavelength of 1550 nm was taken to demonstrate the sensitivity of the probe. The sensitivity was 0.065 nm/(μg/mL) with a linear regression of 0.92. Their results showed a detection limit of 0.001 nm/(μg/mL) and detection range of 0 to 10 µg/mL. Liang et al. [31] demonstrated a microfiber-capillary optofluidic sensor for miRNA-*let7a* detection based on the interference of optical modes. The optofluidic sensor was fabricated by aligning a microfiber in lateral contact with a capillary to form a modal interferometer. With the pre-immobilization of the DNA probe, a log-linear response of 2 nm to 20 μm and a minimum detectable concentration of 212 pm were achieved. Their results showed a detection limit of 2 nM. Zhu et al. [32] demonstrated a free-energy-driven lock/open assembly-based optical DNA sensor. It is a fiber-based fluorescent DNA-sensing platform with immobilized capture probe/microRNA/fluorescent-labeled signal probe (CP/miR/SP) sandwiches formed on the fiber surface. The sensor generates fluorescent signals for quantitative analysis. The developed biosensor was able to detect miR Hsa *let-7a* with a detection limit of 24 pM. Their results showed a detection limit of 24 pM and detection range of 1 pM to 15 nM.

A U-shaped optical fiber is commonly chosen as a biosensor probe based on its sensitivity, compactness, ease of fabrication, and possibly higher compatibility with instrument configurations [33]. In the U-shaped region of a bent fiber, evanescent waves penetrate beyond the thickness of the fiber cladding. The penetration depth of these evanescent waves becomes more pronounced in the outer side of a U-shaped bent fiber depending on the bending radius and wavelength of light. Changes in the surface morphology, diameter, and refractive index of the sensing region that changes in temperature [34], pH [35], as well as the surroundings, affect the U-shaped sensor’s light transmission modes and sensitivity. When the bent region is dipped in a liquid medium, the evanescent waves strongly interact with the liquid medium. Hence, enhanced sensitivity is obtained [36]. U-shaped probes have been demonstrated to have a 10-fold improvement in absorbance sensitivity over straight probes [37]. The use of U-shaped optical fiber as chemical probes seems to be popular over the last two decades, with the fiber used in probes for various chemicals and features of chemicals, including benzene [38], ethanol [39,40,41], toluidine blue [42], pH levels [43], glucose [44,45], and salt [46], but few of studies as a biosensor especially in miRNA of cancer. The sensitive property of U-shaped optical fiber to the external environment is quite suitable for low-concentration bio-detectors analytics. For example, Chandra et al. [47] presented a label-free ultrasensitive U-shaped optical sensor. Human immunoglobulin G (HIgG) was immobilized on this polyaniline coated fiber-optic probe using cross-linked molecules, and goat anti-human immunoglobulin G (GaHIgG) was used as the analytic, the concentration of the analytic was as low as 37 pM. Bharadwaj et al. [48] demonstrated a U-shaped biosensor used as a probe for the label-free detection of *E. coli* at a wavelength of 280 nm. The core diameter and bend radius of this U-bend fiber optic probe were 200 μm and 0.75 mm, respectively. The effective probe of 1 cm was able to detect less than 1000 CFU/mL. The results of the studies reviewed above indicate that bending optical fiber sensors could measure changes in solutions’ refractive index. In fact, researchers have already found that sensors with this design exhibit good sensitivity in solution measurements.

In this study, the application of a sensitive method of U-shaped optical fiber in a miRNA probe is presented. The wavelength shift and transmission loss of this novel tool for the diagnosis of miRNA-133a feature of hepatocellular carcinoma (HCC), i.e., the Mahlavu fragment HCC detector, could be a promising high-sensitive method using a lamping U-shaped optical fiber biosensor. Fiber optic sensors with U-shaped structures have been developed for applications in various physical phenomena and RI detection sensors and to enhance signal sensitivity with controllable cross-sensitivities. We propose a biosensor probe as a detector for miRNA. As mentioned in previous reports, a U-shaped probe used as an evanescent wave absorption sensor possesses high sensitivity as compared with straight and tapered probes. Additionally, it can be used as a point sensor and is less fragile than tapered probes. For the presented fiber, silanization and electrostatic self-assembly were used to bind the gold nanoparticles and miRNA-133a probe onto the glass of fiber surface. For concentration detection of Mahlavu, the transmission loss sensitivity and the coefficient of determination (R^2^) were 27.352 dB/log ng/mL and 0.886, respectively, with a detection limit of 0.0133 ng/mL. The sensitivity and coefficient of the wavelength shift were 67.539 dB/log ng/mL and 0.939, respectively. The developed biosensor could play a crucial role in the prophylaxis, diagnosis, and prognosis of diseases and cancer.

## 2. Theory and Experimental Results

### 2.1. Connection between Wavelength and Refractive Index

In terms of the intermodal interference in a U-shaped SMF, the U-shaped probe consists of a semicircular region with a selected bending radius and straight regions separated by the semicircular one. When the light enters the semicircular region, the light power is split into two portions. A portion of light propagates continuously along with the core as the core mode, another portion of light leaks into the cladding due to the bending, and several cladding modes are excited and propagated along the fiber. At the end of the semicircular region, the cladding modes are coupled back to the core mode, and therefore interference occurs there. Due to the fact that the effective RIs of the cladding mode depends on the RI of the surrounding medium, the transmission spectrum shifts as the surrounding RI changes. The bend induces leakage and transmission loss as well.

Zhang et al. [49] presented the intermodal interference of SMF bending through theoretical and experimental demonstration. After light propagates through the semicircular region of a SMF, the phase difference between the core and the m^th^-order cladding mode is written as Equation (1):

(1) $$\Delta \varphi_{m}\;=\;\frac{2\pi }{\lambda_{D}}\left[n_{co,eff}-n_{cl,m,eff}\right]C\;=\;\frac{2\pi }{\lambda_{D}}\;\Delta n_{eff}\pi R\;=\;\left(2k+1\right)\pi$$

where $n_{co,eff}$ and $n_{cl,m,eff}$ are the effective RIs of the fundamental and the m^th^-order cladding mode, respectively. $\Delta n_{eff}\;=\;n_{co,eff}-n_{cl,m,eff}$ is the effective RI difference between the fundamental mode and the m^th^-order cladding mode, *C* is the length of the semicircular SMF, $\lambda_{D}$ is the wavelength of the transmission dip, R is the bending radius, and *k* is an integer. In this study, the bending diameter, $n_{co,eff}$, and $\lambda_{D}$ were 1.11 mm, 1.4628 and 1550 nm, respectively. Since the effective RIs of the cladding mode depend on the external RI, the transmission dip wavelength shifts when the probe is subjected to external RI perturbation. The sensitivity of the transmission dip to a change in the external RI can be derived from Equation (1) as:

(2) $$\frac{d\lambda_{D}}{dn_{ext}}\;=\;\frac{-\lambda_{D}}{\Delta n_{eff}}\frac{\partial n_{cl,m,gff}}{\partial n_{ext}}/\left[1-\frac{\lambda_{D}}{\Delta n_{eff}}\left(\frac{\partial n_{co,eff}}{\partial \lambda }-\frac{\partial n_{cl,m,eff}}{\partial \lambda }\right)\right]$$

where $\lambda_{D}$ is the loss point of the wavelength in Equation (2). When the refractive index is varied, the effective refractive index of the core mode and cladding mode change accordingly, which makes the wavelength produce a displacement action. As such, we can detect different concentrations due to the wavelength shift caused by this phenomenon.

### 2.2. U-Shaped Fiber Probe Fabrication

**Figure 1:**
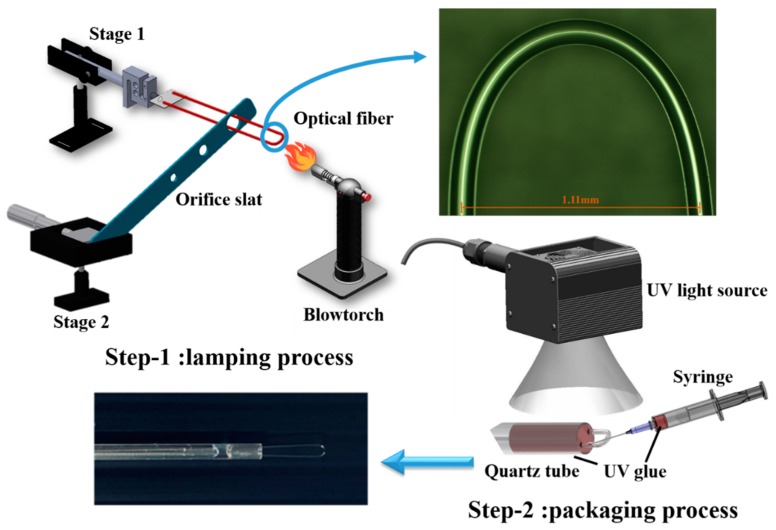
Schematic diagram of fabrication process divided into two steps including the lamping process and packaging process. The optical microscope image shows that the diameter of the semicircular region of fabricated U-shaped optical fiber is 1.11 mm.

The schematic diagram of the fabrication setup is shown in Figure 1. A SMF (Corning SMF28) was chosen to fabricate the U-shaped fiber probe. In Step 1, the present lamping process, their two ends of the optical fiber are put into a hollow glass tube and fixed in the glass tube with acrylic resin, and the orifice slat is used to control the diameter of the U-shaped fiber probe. The diameter of the core and cladding was 125 and 10 µm, respectively, and the bend radius of the optical fiber used was 1.11 mm. The SMF28 fiber consists of all glass and supports single-mode light propagation at a 1310/1550 nm operating wavelength and the effective index of refraction was 1.4682 at a 1550 nm operating wavelength. First, we determined the diameter with a sensitivity optimizing experiment during the glucose concentration test. Wavelength shift and transmission loss were used to compare the sensitivity of U-shaped SMFs with diameters of 1.11 mm (Figure S1) and 1.5 mm (Figure S2).

The results showed that the selected diameter of 1.11 mm had a higher sensitivity in transmission loss of 0.253 dB/% as compared with the diameter of 1.5 mm, which had a transmission loss of 0.0273 dB/%. Thus, the 1.11 mm diameter had high sensitivity because of the larger variation in transmission loss. Second, to obtain a lighter weight detector, the main direction in development was based on a small bending radius, therefore, the 1.11 mm diameter was selected.

The fabrication process was divided into two steps. For the lamping process, the buffer layer of 3 cm in length was stripped from the midpoint of the SMF and, then, the fiber was bent with a larger bending radius. Next, the bent fiber was inserted into a hole of metal orifice slat and both ends of fiber were fixed on a microplatform numbered as Stage 1. The diameter of the hole depended on the final bending radius. The metal orifice slat itself was fixed on the stage numbered as Stage 2. Then, a blowtorch was used to heat the bending region at the top to reduce the bending radius, while the distance between the orifice slat and Stage 1 was carefully adjusted by turning the knob on Stage 2, with pretension stress applied on the fiber. As the glass transition temperature was reached (melting point 800 to 1000 °C), the fiber structure instantly became loose, allowing it to retract into the hole of metal orifice slat. The optical microscope image shows the semicircular region of the fabricated U-shaped optical fiber. As shown in the image, the diameter of the semicircular region was 1.11 mm. The finished U-shaped optical fiber was subsequently inserted into and packaged in a quartz tube to reduce its environmental impact and the effects of human factors, as shown in the photograph. For the packaging process and fine-tuning, UV glue was injected into the front end of the quartz tube using a syringe, and then cured by UV light irradiation. This optical fiber biosensor is quite sensitive to changes in diameter, therefore, to ensure that its diameter remained fixed with high peak-dip contrast it was necessary for it to be packaged.

### 2.3. miRNA-133a Probe Coating

**Figure 2:**
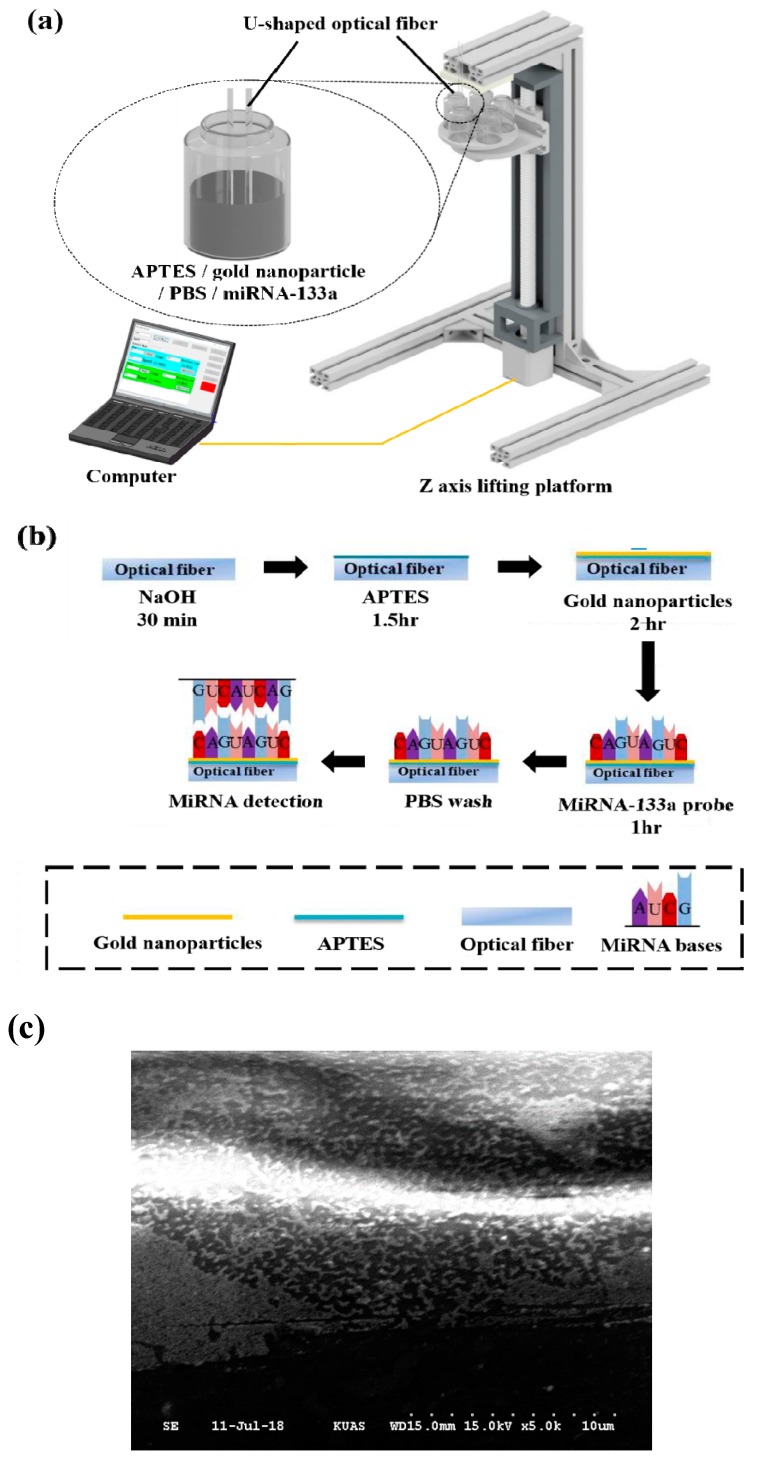
Schematic diagram of miRNA-133a probe coating process. (**a**) Schematic diagram of the experimental setup of miRNA-133a coating; (**b**) procedure of miRNA-133a probe coating; (**c**) SEM image of the optical fiber surface. It clearly shows the fiber surface covered with gold nanoparticles and miRNA-133a.

Figure 2 shows the probe coating process. The packaged U-shaped optical fiber was set on a *Z*-axis lifting platform. The solutions including 5 wt% APTES, a gold nanoparticles solution of citrate ions, phosphate buffered saline (PBS), and a miRNA-133a probe solution were put into individual Vials and placed on a circular rotating table with the *Z*-axis lifting platform, as shown in Figure 2a. The U-shaped optical fiber was immersed into the APTES sample with a specific immersion depth, when the computer-controlled circular rotating table was raised. After the surface modification, the circular rotating table was lowered. Then, the same process was repeated for the gold nanoparticles solution of citrate ions, the PBS, and the miRNA-133a solution. Finally, the optical fiber was rinsed with DI water for 5 min to remove any unbound miRNA-133a probe solution. A commercial surface plasmon resonance (SPR) instrument is bulky and unsuitable for long-distance measurement. Therefore, SPR combined with immunoassay as the main approach was used to improve the sensor’s sensitivity in optical fiber sensing. The sensitivities of these gold nanoparticle sensors were improved further because of the relatively low antigen-antibody binding efficiency.

The details of the miRNA-133a probe binding procedure are shown in Figure 2b. After being washed by DI water, the optical fiber was immersed into 40% (*v/v*) NaOH for 30 min to generate hydroxyl groups on the fiber surface. Next, it was immersed into 5 wt% APTES for 1 h to produce amine groups that linked with the hydroxyl groups on the surface of fiber for silanization. Then, the fiber was immersed into the gold nanoparticles solution with citrate ions for 2 h, thus, the gold nanoparticles were electrostatically adsorbed by the amine groups for the self-assembly process. The fiber was subsequently immersed in the miRNA-133a probe solution for 1 h. Figure S3 and Figure 2c show the scanning electron microscope (SEM) images of the surface of the semicircular region. It can be clearly seen that the surface is covered with gold nanoparticles and miRNA-133a. A chemical bond was formed between a functional group of biomolecules and the amino group of the linker to immobilize biomolecules covalently cell line, and therefore they bind with the gold nanoparticles to the surface of optical fiber. Finally, the miRNA-133a probe was extracted from the relevant, and PBS was used to wash out the unbound miRNA-133a probe.

### 2.4. Experimental Setup

The U-shaped fiber sensor was set on a *Z*-axis lifting platform. One of the U-shaped fiber sensors was connected to a superluminescent diode (SLD) light source, and the other was connected to an optical spectrum analyzer (OSA). In this study, there were three types of cells used in the experiment, including hepatoma cell lines Mahlavu, SK-*Hep1*, and standard miRNA-133a samples. The specimen solutions were diluted to a series of concentrations and poured into microtubes, and then placed into a dry bath incubator sequentially. In this experiment, we used U-shaped optical fibers with the pH controlled at approximately 7.4; the temperature varied from 40 to 90 °C during the sensing process. It is important to note that all of the specimens were stored in a double-stranded structure and were denatured at 90 °C for detection. For one operating cycle, the heating process lasted for 30 min and the cooling process for 50 min. Then, the specimens were recovered in the double-stranded structure at 40 °C. The developed sensor was immersed in the heated specimens when their temperature was 90 °C. The light excited by the SLD was transmitted into one end of the fiber sensor and propagated along the fiber, and was absorbed finally by the OSA. The numerical data of the given light signal was, then, processed by the LabVIEW software, and the Grapher software was used to plot the associated spectrum diagrams.

## 3. Results and Discussion

**Figure 3:**
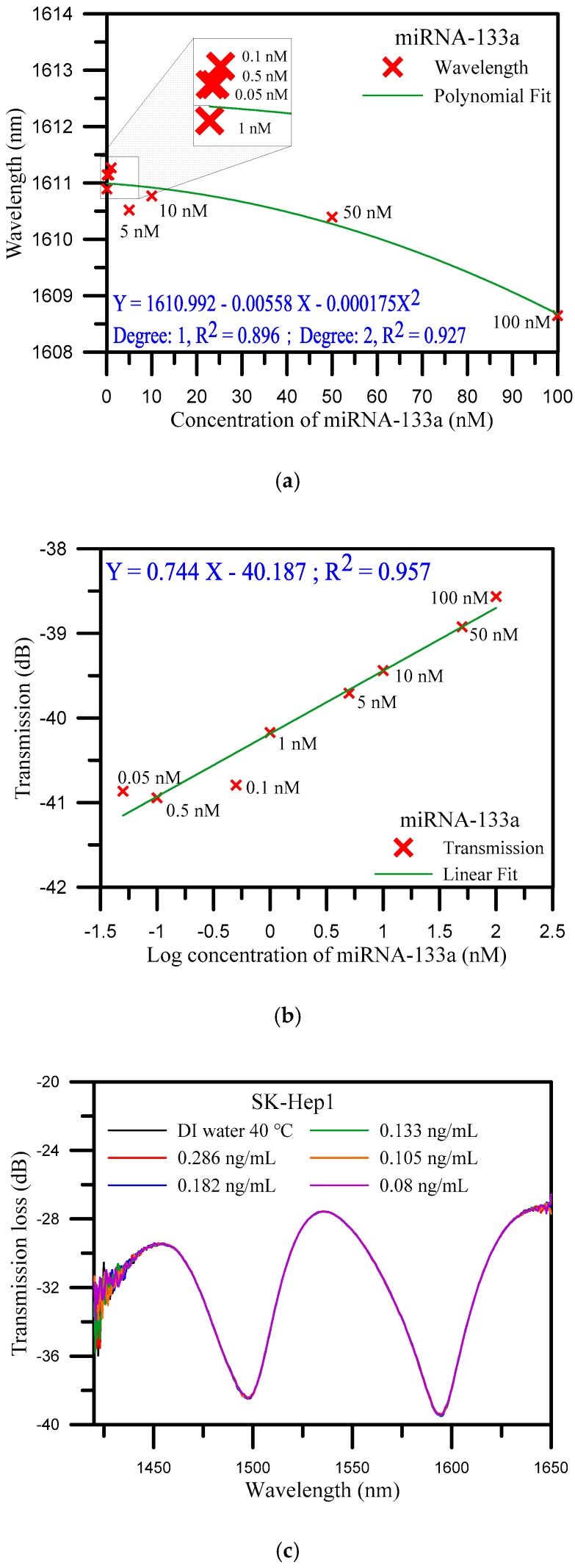
(**a**) Wavelength shift analysis diagram of standard miRNA-133a sample detection; (**b**) analysis diagram of transmission loss for standard miRNA-133a sample detection; and (**c**) spectrum diagrams of various concentrations of SK-*Hep1* specimen solution.

In order to prove the functionality of the fabricated biosensor, the standard sample of miRNA-133a was detected. The standard sample solution with a concentration of 100 μM was diluted to a series of concentrations including 100, 50, 10, 5, 1, 0.5, 0.1, and 0.05 nM. Standard samples of miRNA-133a, in this study, are prepared from a high concentration to a low concentration, whereas standard samples of miRNA-133a are from a low concentration to a high concentration for detection. As the concentration changes from 0.05 nM to 100 nM, the wavelength is changed from 1610.895 nm to 1608.646 nm, that is, the wavelength shifted from a longer wavelength to a shorter wavelength, with a deviation of 2.249 nm. The deviation of the transmission loss was 2.302 dB. Figure 3a illustrates the analysis of the wavelength shift instead of a polynomial function. The method is based on a polynomial fit of the shift of wavelength versus the concentration of miRNA-133a that obtained the coefficient of determination (R^2^) at 0.927 under degree two and at 0.896 under degree one. Figure 3b shows the analysis diagram of the transmission loss. The transmission loss increased from −38.565 to −40.867 dB, as well as the concentration of miRNA-133a decreased. The sensitivity of the transmission loss was 0.744 dB/log nM with R^2^ = 0.957, with a detection limit of 0.5 nM. Due to the fact that the effective RIs of the cladding mode depend on the RI of the surrounding medium, the transmission spectrum shifts as the surrounding RI changes according to Equation (2). The transmittance is a function of the coupling coefficient for the cladding mode, which is related to the amplitude of the refractive index change, owing to the surrounding RI changes. The transmission loss of the spectrum increases as the concentration of miRNA-133a decreases which can be ascribed to changing the external RI changes. These test results showed that the functionality of the developed biosensor is good.

SK-*Hep1* is an immortal, human cell line derived from the ascetic fluid of a patient with adenocarcinoma of the liver [50]. Due to the absence of miRNA-133a gene fragment in the SK-*Hep1* specimen, it was used as a control in this study. The SK-*Hep1* was diluted to a series of concentrations from 0.286 to 0.08 ng/mL. Figure 3c shows the spectra of transmission loss and wavelength by various concentrations. As was anticipated, there was no variation in the diagrams. These results demonstrated that the U-shaped optical fiber was highly specific and sensitive to the miRNA-133a gene fragment as compare with results with SK-*Hep1*.

The miRNA-133a probe is employed as a tool to detect hepatoma cell lines Mahlavu, due to the gene fragment of miRNA-133a existing in Mahlavu cell line specimens. In this study, therefore, a Mahlavu cell line specimen solution with a concentration of 2000 ng/mL was diluted.

**Figure 4:**
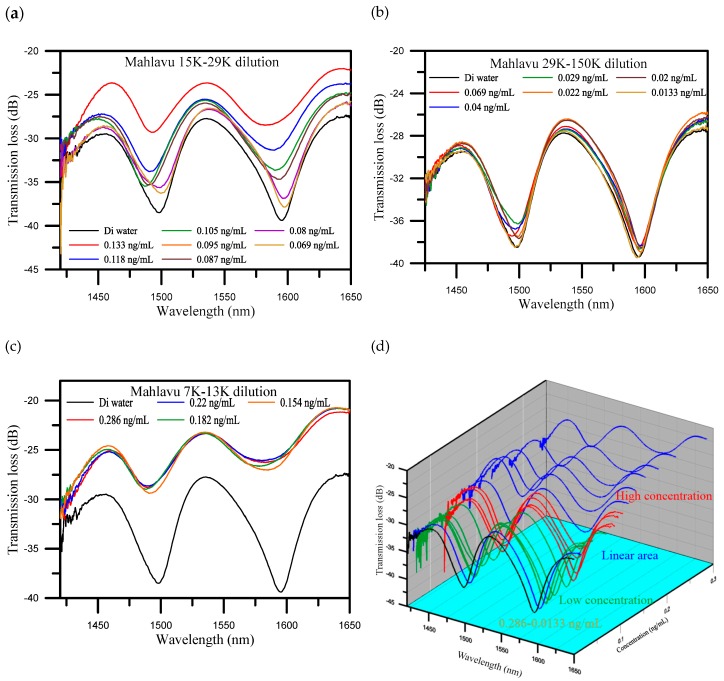
Spectrum diagrams of different concentrations of Mahlavu specimen solutions which were divided into three areas. (**a**) The high concentration area; (**b**) the linear area; (**c**) the low concentration area; and (**d**) three-dimensional (3D) spectrum diagrams of all concentrations.

The distribution of the concentrations of the diluted Mahlavu specimen samples ranged from 0.286 to 0.0133 ng/mL. Referring to the experimental results, the distribution of the concentrations was divided into three areas. They included a saturation area or high concentration area (from 0.286 to 0.154 ng/mL), a linear area (from 0.133 to 0.069 ng/mL), and a low concentration area (from 0.069 to 0.0133 ng/mL). Figure 4 illustrates the spectrum diagrams of the different concentrations distributed in these three areas. The wavelength shifted from 1583.158 to 1585.032 nm and the transmission loss increased from −26.273 to −27.026 dB, when the concentration decreased in the high concentration area (Figure 4a). These results indicate that the variations in both the wavelength and transmission loss were not conspicuous. For the linear area (Figure 4b), the wavelength shifted from 1581.909 to 1594.903 nm, with an increase of 12.994 nm. The transmission loss was also increased, from −28.502 to −38.506 dB, with a variation of 10.004 dB. It is noteworthy that it presented good sensitivity to the variation of concentration in the linear area. Finally, for the low concentration area (Figure 4c), the wavelength shifted from 1594.903 to 1594.028 nm, and the transmission loss increased from −38.506 to −39.483 dB. These results show that the variations in both wavelength and transmission loss were not conspicuous for high and low concentration areas. As shown in Figure 4c, we can obviously see that the spectrum with the concentration of 0.0133 ng/mL closed with the one for DI water, the detection limit was found to be 0.0133 ng/mL.

**Figure 5:**
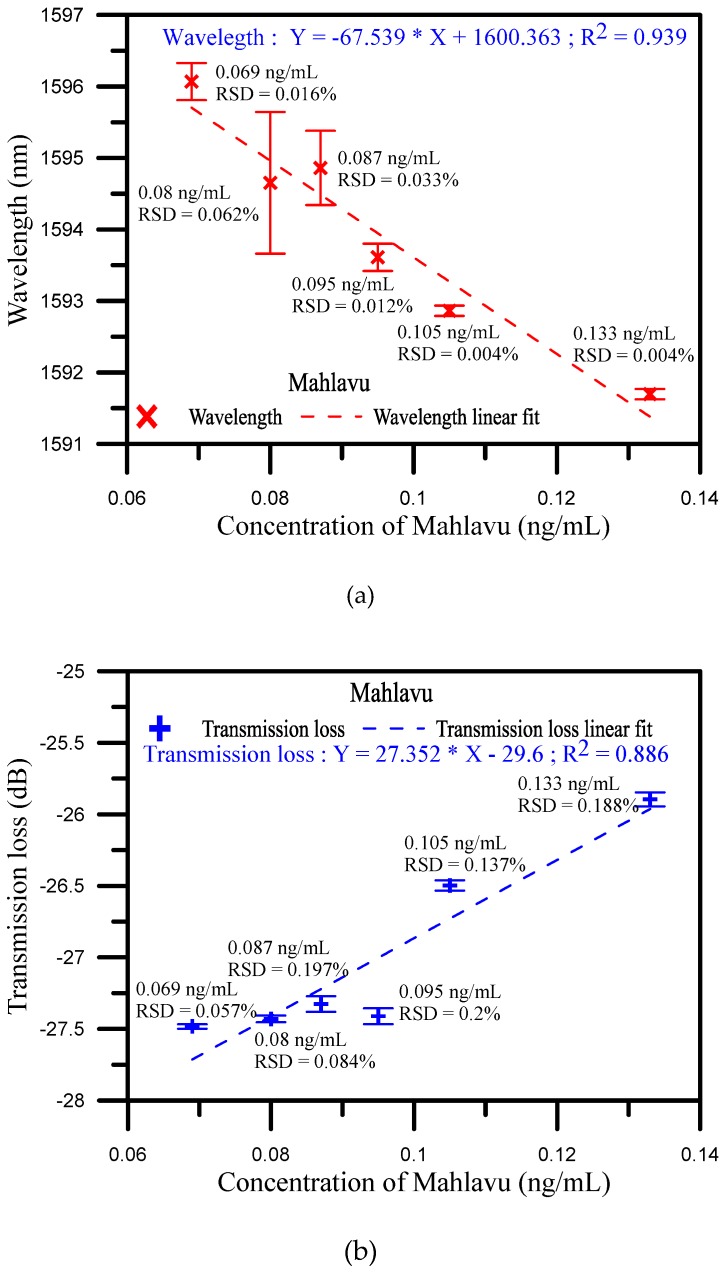
(**a**) Wavelength shift analysis diagram of Mahlavu specimen detection for the middle concentration area; (**b**) Transmission loss shift analysis diagram of Mahlavu specimen detection for the middle concentration area.

Figure 5a,b presents the wavelength shift and transmission loss analysis of the Mahlavu specimen detection, respectively. As mentioned before, in the high and low concentration areas, the detection of wavelength variations were not obvious. In contrast, as shown in Figure 5, the sensitivity of the wavelength shift and transmission loss and linearity were good in the middle area. The reflection resonance wavelength of U-shaped appears with transmission loss, and the resonance condition is generated by the coupling between the propagating core mode and the leakage-propagating core mode. Therefore, the sensitivity of the analysis is increased when the wavelength shift and transmission loss are analyzed together with controllable cross-sensitivities. For the analysis of the wavelength shift instead of a polynomial function, the method is based on a polynomial fit of the shift of wavelength versus the concentration of Mahlavu that obtained R^2^ = 0.914 at degree two and R^2^ = 0.839 at degree one between 0.069 and 0.133 ng/mL. This phenomenon was also observed in the transmission loss analysis shown in Figure S5. The variations in the transmission loss were also not observed in both high and low concentration areas (as shown in Figure S5b). In contrast, in the middle area, the transmission loss sensitivity of determination was 27.352 dB/log ng/mL, the sensitivity and coefficient of the wavelength shift were 67.539 dB/log ng/mL and 0.939, respectively, indicating excellent sensitivity to variations in the Mahlavu specimen concentration (as shown in Figure S5c). Compared with the reported works [28,30,31] (as shown in Table S1), the detection limits and detection range have improved. We also finished a three-cycle experiment to detect the Mahlavu fragment. The results are shown in the following Figure S4. The spectrum diagrams of the different concentrations in each cycle are nearly the same, which proves that the proposed sensing probe possesses good repeatability. For each concentration in one cycle, we used the same probe for detection, and used PBS to regenerate another beginning fiber sensor between different concentration measurements. Figure S3 shows the SEM image of the sensor surface. We can clearly see that the miRNA-133a specimens were bound to the probe surface. The goal of our study was to also present a new application of U-shaped optical fibers to detect the Mahlavu fragment, which would be of substantial value for prophylaxis, diagnosis, and prognosis of diseases, including cancer.

## 4. Conclusions

We provided a tool for miRNA-133a of hepatocellular carcinoma (HCC) feature diagnosis that could be promising, using nanoparticle markers on a U-shaped optical fiber. This study demonstrated the use of a U-shaped optical fiber as a novel biosensing tool to detect the feature of Mahlavu in hepatocellular carcinoma (HCC). Specifically, the U-shaped optical fiber was used as a biosensor for microRNA detection. It was easily fabricated by the lamping process and packaged in a quartz tube to eliminate human negligence. In addition, silanization and electrostatic self-assembly were used to bind gold nanoparticles and the miRNA-133a probe onto the fiber surface. In order to prove the functionality of the developed biosensor, experiments were conducted with three types of specimens, including Mahlavu, SK-*He1*, and miRNA-133a standard samples. In the miRNA-133a detection, the sensitivity of the transmission loss was 0.744 dB/log nM with R^2^ = 0.957 and a detection limit of 0.5 nM. The SK-*Hep1* served as a control, and therefore there was no variation in the spectrum. These results showed that the developed fiber biosensor is highly specific to the miRNA-133a gene fragment. Moreover, for the Mahlavu fragment detection results, the R^2^ = 0.914 at degree two, and R^2^ = 0.839 at degree one for the wavelength shift was based on a polynomial fit. The sensitivity and linear regressions of the wavelength shift were 27.352 dB/log ng/mL and 0.886, respectively, while the detection limit was 0.0133 ng/mL. These results showed that the developed biosensor provides excellent sensitivity for Mahlavu specimen detection. The experimental results also showed that the transmission loss decreased as the concentration of the surrounding medium increased, consistent with the theoretical description. Therefore, the developed biosensor could be valuable for clinical miRNA detection and has high potential for application in the prophylaxis, diagnosis, and prognosis of diseases and cancer.

## Supplementary Materials

The following are available online at https://www.mdpi.com/1424-8220/20/5/1509/s1, Figure S1: Wavelength and transmission loss analysis for glucose by U-shape diameter at 1.11 mm, Figure S2: Wavelength and transmission loss analysis for glucose by U-shape diameter at 1.5 mm, Figure S3: SEM image of the biosensor surface, Figure S4: Three cycles of spectrum of the Mahlavu assay with U-shaped optical fiber, Figure S5: Analysis of transmission loss of Mahlavu assay in three cycles. Table S1: Comparison between this study and reported works on optical fiber sensors.

## Abbreviations

APTES	3-aminopropyl-triethoxysilane solution
DI	Deionized
DNA	deoxyribonucleic acid
LPFG	long-period fiber grating
OSA	optical spectrum analyzer
PBS	phosphate buffered saline
PM	polarization maintaining
PNA	peptide nucleic acid
SEM	scanning electron microscope
SLD	superluminescent diode
SMF	single-mode fiber
SPR	surface plasmon resonance
TFBG	tilted fiber Bragg grating
UV	Ultraviolet
