# Supplementary material for: A Lamping U-Shaped Fiber Biosensor Detector for MicroRNA
Source: Sensors (Basel). 2020 Mar 9;20(5):1509. doi: 10.3390/s20051509 (PMC7085725; doi:10.3390/s20051509)
Supplement: Supplementary file 1 [file sensors-20-01509-s001.pdf]

sensors

Supporting Information

A Lamping U-Shaped Fiber Biosensor Detector for MicroRNA

Hsin-Yi Wen [1], Chun-Wei Huang [1], Yu-Le Li [1], Jing-Luen Chen [1], Yao-Tsung Yeh [2] and Chia-Chin Chiang [1,*]

[1] Department of Mechanical Engineering, National Kaohsiung University of Science and Technology, 415 Chien Kung Road, Kaohsiung 80778, Taiwan; hywen@nkust.edu.tw (H.-Y.W.); cwhuang@kuas.edu.tw (C.-W.H.); love02111110@gmail.com (Y.-L.L.); ChineseTaipeibasketball@gmail.com (J.-L.C.)

[2] Department of Medical Laboratory Science and Biotechnology, Fooyin University, Kaohsiung 83102, Taiwan; glycosamine@yahoo.com.tw

* Correspondence: ccchiang@nkust.edu.tw

Received: 11 February 2020; Accepted: 6 March 2020; Published: date

We chose a diameter of 1.11 mm for the U-shaped optical fiber's semicircular region for the following reasons. First, we determined the diameter with a sensitivity optimizing experiment during the glucose concentration test. The experiment compared the wavelength shift and transmission loss when the region's diameter was 1.11 mm (**Figure S1**) and 1.5 mm (**Figure S2**). The results showed that the present diameter of 1.11 mm has a higher transmission loss of 0.253 dB/% compared to the diameter of 1.5 mm, which has a transmission loss of 0.0273 dB/%. Thus, the 1.11 mm diameter has high sensitivity because of the larger variation in transmission loss. Second, to obtain a lighter weight detector, the main direction in development is based on a small bending radius. Therefore, the 1.11 mm diameter was selected.

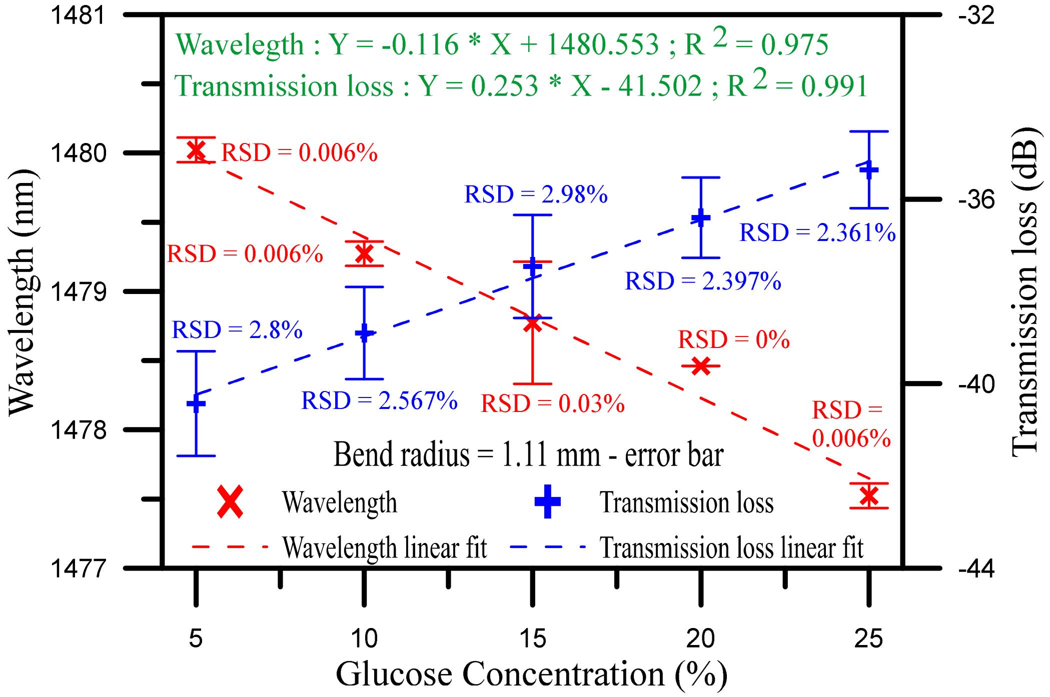

Figure S1. Two cycles spectrum diagrams of analysis chart of wavelength and transmission spectra during the glucose concentration test of the diameter of the semicircular region of 1.11 mm. That the

maximum wavelength sensitivity of 0.116 nm/%, coefficient of determination of R^2=0. 0.975; the transmission loss sensitivity of 0.253 dB/%, coefficient of determination of R^2=0.991。.

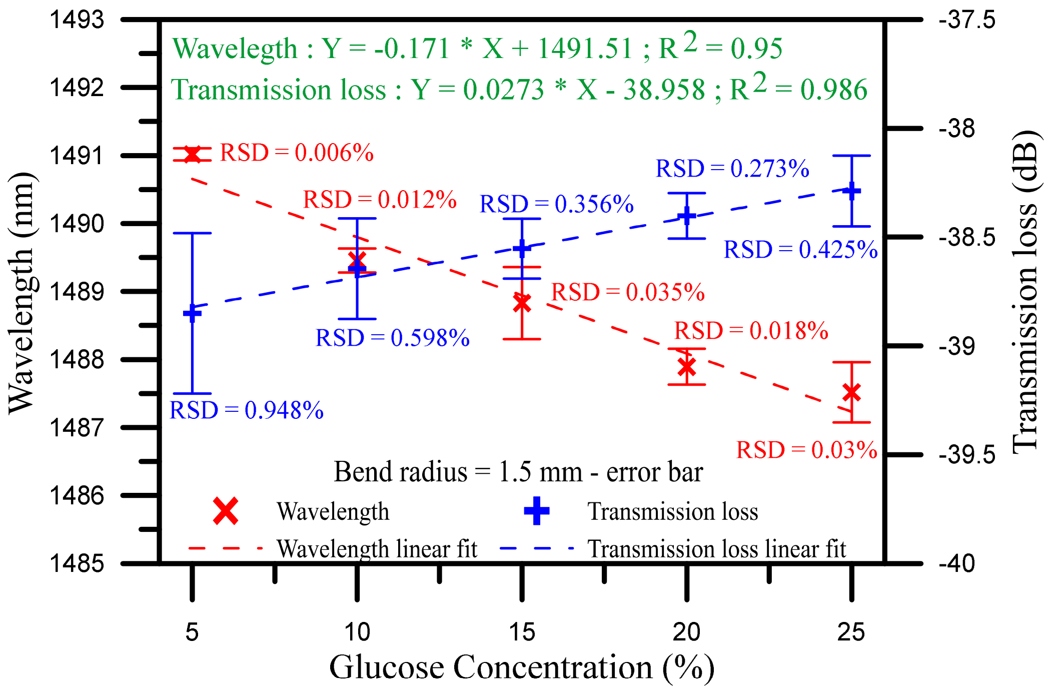

Figure S2. Two cycles spectrum diagrams of analysis chart of wavelength and transmission spectra during the glucose concentration test of the diameter of the semicircular region of 1.5 mm. That the maximum wavelength sensitivity of −0.171 nm/%, coefficient of determination of R^2 = 0.95; the transmission loss sensitivity of 0.0273 dB/%, coefficient of determination of R^2 = 0.986.

Figure S3 shows the scanning electron microscope (SEM) images of the surface of the semicircular region.

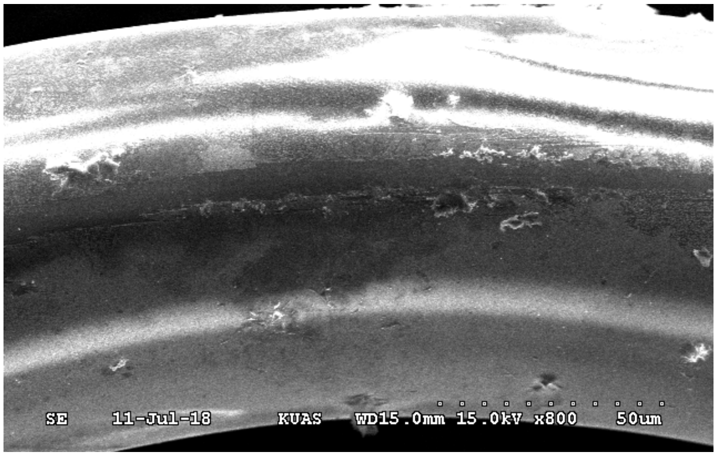

Figure S3. SEM image of the biosensor surface. The gene fragments of miRNA-133a specimens are clearly bound to the probe surface.

We conducted a 3-cycle experiment. The results are shown in the following **Fig. S4**. The spectrum diagrams of the different concentrations in each cycle are nearly the same, which proves that the proposed sensing probe possesses good repeatability.

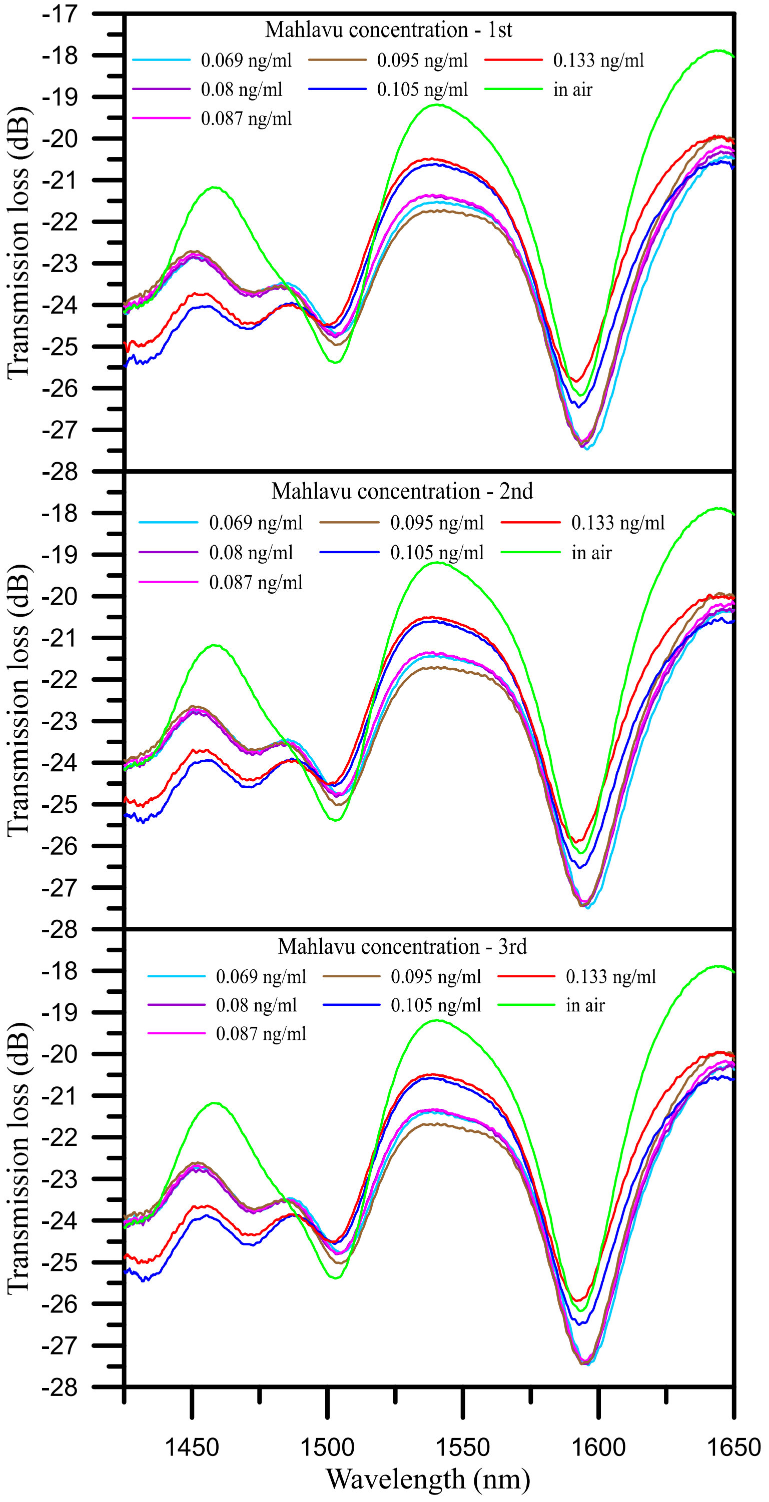

Figure S4. Three cycles of spectrum diagrams of the different Mahlavu fragment concentrations with U-shaped optical fiber miRNA-133a probe was employed.

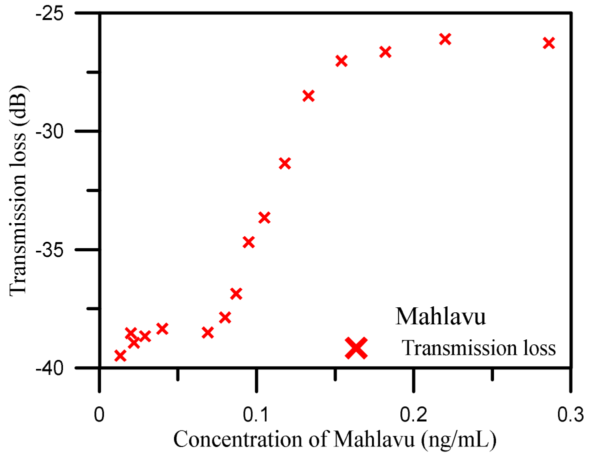

(a)

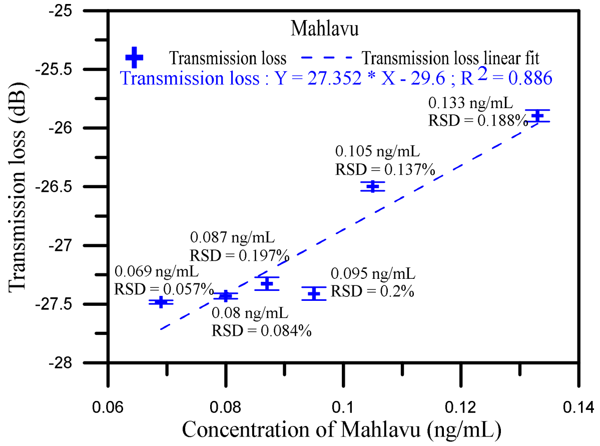

(b)

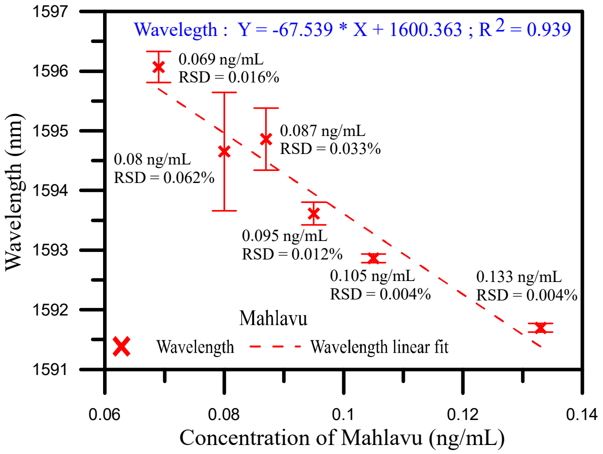

(c)

Figure S5. Analysis diagram of transmission loss of Mahlavu specimen detection for (a) all concentrations and; (b) transmission loss of the middle area in three cycles; (c) wavelength shift of the middle area in three cycles.

We compared with the other reported works and modified sentence in the revised manuscript. We also compared with the following reported works and combine the results of the comparison into **Table S1**.

Table S1. Comparison between this study and reported works on optical fiber sensors.

Authors	Measuring Range	Maximum Sensitivity	R^2	Limit	Year	Ref.
**This study**	0.286 to 0.0133 ng/ml	−763.916 nm/ng/ml<br>−0.022 nm/nM	0.939	0.5 nM<br>0.0133 ng/ml		
**Xianfeng Chen *et al.***	1 μM	0.254 nm/μM			2007	[26]
**Michele Sozzi *et al.***	120 nM	0.01 nm/nM			2011	[27]
**Alessandro Candiani *et al.***	10 nM、100 nM			10 nM	2011	[28]
**Yunyun Huang et al.**	1 nM to 1 μM	2.393 nm/M	0.985	0.1 nM	2015	[29]
**S.-H. Hsu *et al.***	0 to 10 μg/mL	0.064 nm/(μg/mL)	0.92	0.001nm /（μg/ ml）	2016	[30]
**Lili Liang *et al.***	20 μM	0.105 nm/μM		2 nM	2017	[31]
**Xiyu Zhu *et al.***	1 pM to 15 nM			24 pM	2017	[32]

© 2020 by the authors. Submitted for possible open access publication under the terms and conditions of the Creative Commons Attribution (CC BY) license (http://creativecommons.org/licenses/by/4.0/).
